# Modern surgical treatment of breast cancer

**DOI:** 10.1016/j.amsu.2020.06.016

**Published:** 2020-06-23

**Authors:** M. Riis

**Affiliations:** a Department of Oncology, Section of Breast- and Endocrine Surgery, Oslo University Hospital, Oslo, Norway; b Department of Cancer Genetics, Institute for Cancer Research, The Norwegian Radium Hospital, Oslo University Hospital, Oslo, Norway

**Keywords:** Breast cancer, Surgical treatment of breast cancer, Oncoplastic surgery, Surgical algorithms

## Abstract

Breast cancer is the most frequent cancer in women all over the world. The prognosis is generally good, with a five-year overall survival rate above 90% for all stages. It is still the second leading cause of cancer-related death among women. Surgical treatment of breast cancer has changed dramatically over the years. Initially, treatment involved major surgery with long hospitalization, but it is now mostly accomplished as an outpatient procedure with a quick recovery. Thanks to well-designed retrospective and randomly controlled prospective studies, guidelines are continually changing. We are presently in an era where safely de-escalating surgery is increasingly emphasized. Breast cancer is a heterogenous disease, where a “one-size-fits-all” treatment approach is not appropriate. There is often more than one surgical solution carrying equal oncological safety for an individual patient. In these situations, it is important to include the patient in the treatment decision-making process through well informed consent. For this to be optimal, the physician must be fully updated on the surgical options. A consequence of an improved prognosis is more breast cancer survivors, and therefore physical appearance and quality of life is more in focus. Modern breast cancer treatment is increasingly personalized from a surgical point of view but is dependent on a multidisciplinary approach. Detailed algorithms for surgery of the breast and the axilla are required for optimal treatment and quality control. This review illustrates how breast cancer treatment has changed over the years and how the current standard is based on high quality scientific research.

## Highlights

• Breast cancer is a heterogenous disease which requires updates on scientific research to offer patients the best possible and personalized treatment.
• The extent of surgery of the breast and the axilla has advanced considerably over the years as a result of translational research and clinical studies. The collaboration between specialties across different countries is needed to exchange experience and improve international guidelines.
• Multidisciplinary teams should be established in all units involved in treatment of breast cancer patients. In the consultation with the patient it is important to inform the patient of all treatment options for the patient to be able to make an informed consent.
• The major trend in surgical treatment of breast cancer is de-escalating surgery and more focus on tumor biology.

## 1 Introduction

### 1.1 Epidemiology

Breast cancer is by far the most frequent cancer among women today. In 2018, there were 2 088 849 new cases worldwide representing 11.6% of all new cancers diagnosed that year. In the same period, 626 679 patients died of breast cancer, which was 6.6% of all cancer related deaths, making it the second most common cause of cancer-related death after lung cancer [1]. In the USA, the incidence and mortality in 2018 were 268 670 and 62 330, respectively [2]. In 2019, although the incidence in the USA increased to 271 270 in 2019, the estimated breast cancer mortality was reduced to 42 260 [3]. In the UK the incidence is around 55 200 with approximately 11 400 breast cancer deaths [4]. There is a higher incidence rate in Western nations, but a higher mortality rate in less developed countries [1]. In all age groups, black women are generally diagnosed at a more advanced stage and have higher mortality rates than other racial/ethnic groups around the world. This can be explained by intrinsic biological differences in lymph node metastasis, distant metastasis, and the prevalence of triple-negative (TN) tumors in different racial groups. TN tumors are those that do not express hormone receptors (HR) or overexpress HER2 on the surface of the breast cancer cells [5].

### 1.2 Treatment - overview

**Fig. 1:**
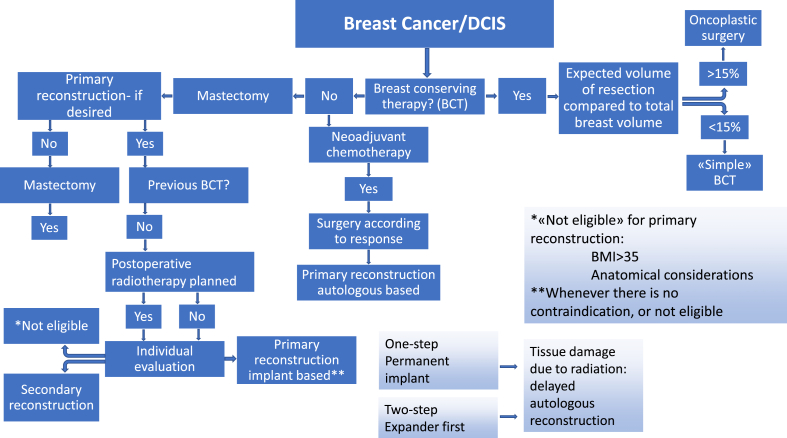
A flowchart illustrating the current guidelines in Norway (https://www.helsebiblioteket.no/retningslinjer/brystkreft/kirurgisk-og-kurativ-behandling/kirurgisk-taktikk-og-teknikk/flytskjema-for-brystkirurgiske-alternativ). There are many steps. Most decisions are made by a multidisciplinary team consisting of radiologists, pathologists, oncologists, and breast surgeons. In some cases, there are plastic surgeons involved. It is important to include the patients in the decisions in cases where the different available options are equivalent in terms of prognosis.

**Fig. 2:**
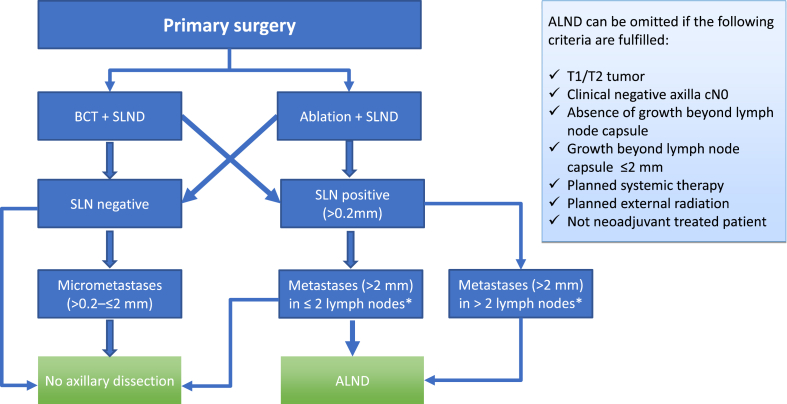
Flowchart for surgery in the axilla in cases where patients are treated with primary surgery. BCT, breast conserving therapy. SLND, sentinel lymph node dissection. SLN, sentinel lymph node. ALND, axillary lymph node dissection (Burstein, Curigliano et al., 2019).

**Fig. 3:**
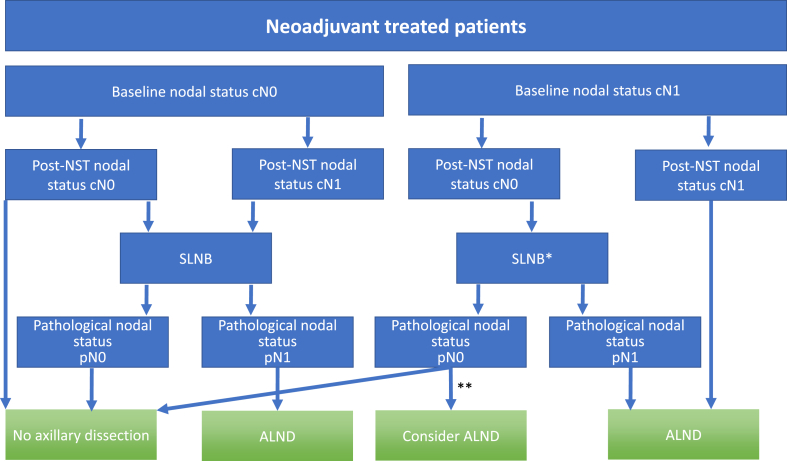
Flowchart for surgical treatment of the axilla in neoadjuvant treated patients. cN, clinical nodal status. pN, pathological nodal status. SLNB, sentinel lymph node biopsy. ALND, axillary lymph node dissection. NST, neoadjuvant systemic therapy. Patients with pN2 or pN3 are advised to have a ALND. * SLNB with certain recommendations; SLNB + > 2 resected lymph nodes. Dual tracing. Histological examination with H&E and IHC. Metastases >0,2 mm warrant ALND. In some institutions targeted axillary dissection (TAD) is advised. ** In these cases, ALND can be omitted provided the above recommendations. When there is doubt, ALND is advised (Burstein, Curigliano et al., 2019).

Treatment of breast cancer has changed over the years, both surgically and medically. The intention of surgical treatment is to achieve local control, prevent locoregional recurrence and improve survival [6,7]. The different surgical approaches for treating breast tumors include mastectomy alone or with reconstruction, either primary or delayed, or breast conserving therapy (BCT), with or without the use of oncoplastic techniques (Fig. 1). The extent of axillary surgery is a continuous subject of discussion. The use of sentinel node diagnostics is standard, with or without subsequent complete axillary dissection. In selected cases, direct complete axillary dissection is recommended [6,7] (Fig. 2, Fig. 3).

## 2 Surgical treatment of the breast

### 2.1 Breast conserving therapy (BCT)

BCT can be performed with a simple wide excision or with different levels of oncoplastic surgery. For a selection of breast cancer patients, BCT is considered the perfect surgical option, and is oncologically safe [[8], [9], [10]]. Oncoplastic BCT facilitates larger resections in relatively small breasts but was introduced mainly to improve cosmetic outcome as up to 40% of women undergoing BCT report poor cosmetic outcome [11]. There are several different factors that may influence cosmetic outcome, both patient-related and surgeon-related, but the most important is the volume of breast tissue that needs to be excised [12]. Locoregional recurrence was previously considered to be a surgical failure but meta-analyses have illustrated that tumor biology is more important in terms of prognosis [13,14].

The safety of oncoplastic surgery has been reviewed by Campbell et al. [15]. Comparing conventional BCT with oncoplastic BCT based upon earlier studies is challenging because the cohorts often differ significantly. The safety of conventional BCT is often based on cohorts with smaller tumors [16,17], while studies focusing on oncoplastic BCT tend to evaluate patients with larger tumors, with or without neoadjuvant treatment [[18], [19], [20], [21], [22], [23], [24]]. Women with large tumors and concomitant large, pendulous breasts are a major challenge for conventional BCT, but they can be treated by reduction mammoplasty with good oncological and cosmetic outcomes [[23], [24], [25], [26]]. Prospective randomized studies are difficult to initiate for obvious ethical reasons and the best way to establish treatment safety is via comparative observational studies and systematic reviews. There are currently published results from eight comparative studies focusing on recurrence rates and survival, which are the best measures of oncologic safety [10,19,[27], [28], [29], [30], [31], [32]]. Their results show non-inferior outcomes for different levels of oncoplastic surgery compared with standard BCT. Due to size differences in individual studies, evidence supporting oncoplastic BCT should be compared to mastectomy rather than to conventional BCT [10,33].

Another bias in these comparative studies is the extent of oncoplastic surgery, which is not clearly defined in the individual studies according to the techniques described by Clough et al. [18]. There are two case-matched retrospective studies from a single institution comparing oncoplastic BCT with conventional BCT and mastectomy in breast cancer patients diagnosed between 2000 and 2008 [30,31]. These include a large series of patients with matched control groups and their results are therefore considered adequate evidence of the safety of oncoplastic BCT. The first study included approximately 500 patients who had undergone oncoplastic BCT and twice as many who had undergone conventional BCT [30]. There was no difference in the histopathological characteristics between the two groups but there was more multifocal disease in the oncoplastic BCT group. Overall survival (OS) at 10 years was similar (91.4% for oncoplastic BCT vs 91.3% in conventional BCT). The oncoplastic BCT group experienced a slightly higher incidence of local recurrence (LR) both at 5 years (3.2% vs 1.8%) and 10 years (6.7% vs 4.4%) but this was not statistically significant. Regional and distant events were similar between the two groups [30]. The second study was nearly identical except that oncoplastic BCT was compared to mastectomy with around 200 patients in the oncoplastic BCT group and twice as many in the mastectomy group [31]. Results were similar for OS (87.3% in the oncoplastic BCT group and 87.1% in the mastectomy group), and for disease free survival (DFS) (60.9% in the oncoplastic BCT group and 56.3% in the mastectomy group). There was a slight increase in the incidence of regional recurrence (RR) in the mastectomy group compared to the BCT group (7.3% vs 3% at 10 years), while the opposite was the case for local events; however, the differences were not statistically significant. Clearly, more evidence is needed to support the level of oncological safety and improved esthetic outcome of oncoplastic surgery [15]. There is global agreement on the urgent need for prospective multicenter studies to optimize patient selection and for standardized criteria to qualify and accredit oncoplastic surgical training centers [15,34,35].

Regardless of the surgical techniques employed, tumor-free surgical margins are mandatory. These negative margins have no tumor cells in the ink-colored section, the so-called “no tumor on ink” guideline. The guidelines concerning margins have changed over the years, but today “no tumor on ink” is considered adequate [6,7,[36], [37], [38]]. Wider margins do not have a positive impact in terms of LR in invasive breast cancer [39]. In ductal carcinoma *in situ* (DCIS) a margin of 2 mm is recommended to reduce the risk of locoregional recurrence; however, minimum margin distances >2 mm are not significantly associated with a further reduction in the likelihood of LR in women receiving radiation [40,41]. Involved surgical margins occur in 20%–40% of patients undergoing BCT which means they need a second surgical procedure [11,42]. This can potentially delay adjuvant therapy and is associated with increased stress and anxiety in the patients. Oncoplastic BCT allows a wider resection and involved margins are less frequent. This facilitates the timing of adjuvant treatment and is also positive from a cosmetic perspective [39,43,44].

Campbell et al. [15] compared 13 studies analyzing resection margins in conventional BCT and oncoplastic BCT [19,[27], [28], [29], [30],^32^,^33^,[44], [45], [46], [47], [48], [49]], and in eight of these studies oncoplastic BCT had a superior outcome [[27], [28], [29],[44], [45], [46], [47],^49^]. A population-based study of 7303 eligible women with stage I and II breast cancers was performed in the United States [50]. It was designed to specifically address surgical margins and the attitude of the individual surgeons in this respect. Between 2013 and 2015, the number of BCT procedures increased by 13% (p = 0.002), and the number of mastectomies was correspondingly reduced. Re-excision was reduced following implementation of the “no tumor on ink” guidelines [50], and this reduction was more pronounced in those hospitals with the most experienced surgeons [50]. As for breast cancer treatment in general, tumor morphology is relevant when considering involved margins. A significant number of invasive lobular carcinomas are found to have positive surgical margins and are in need of re-excision [51]. A possible delay to adjuvant treatment is an important issue when considering the choice of surgical procedure. In addition to involved margins, complications such as postoperative infections and bleeding can influence further treatment; however, when comparing conventional BCT, oncoplastic BCT, and mastectomy, there were no significant differences in terms of complications [29,45]. Furthermore, comparing studies confirmed that regardless of the surgical procedure selected there is no significant difference in the delay to adjuvant treatment [20,24,52].

The range of patients receiving BCT as well as the range of patients in need of only one surgical procedure varies according to the experience of the surgeon. This is illustrated in an American survey considering surgical margins and reoperation rates [50]. This was a population-based survey of approximately 7000 eligible patients, which was eventually reduced to 3279 in the analytic cohort. The 488 surgeons treating these patients were asked to participate in a survey on margins after lumpectomy and 342 responded in full. Those treating more than 50 breast cancers annually were significantly more likely to report a “no tumor on ink” margin as adequate (85%; n = 105) compared with those treating 20 cases or fewer (55%; n = 131) (P < 0.001) [50].

There is an ongoing debate as to whether breast cancer surgery should be centralized, and statistics showing the different procedures and the varying results from different surgical institutions support this [50,53]. EUSOMA (European Society of Breast Cancer Specialists) established a working group to define the requirements for a Europe-wide, high quality, specialist service for breast cancer and other benign diseases of the breast [54]. In addition to detailing skills in surgery, radiology, pathology, and oncoplastic surgery, there are strict requirements that must be met by the breast centers themselves. The European guidelines recommend treating at least 150 new cases per year for every 250 000 inhabitants [35,[55], [56], [57], [58], [59]], and the core team should meet the requirements in terms of composition and specialist training.

### 2.2 Mastectomy

A mastectomy is no longer a straightforward procedure. It can be performed as a conventional mastectomy, which is often a good choice of treatment for a certain group of patients [60], as it can be performed as an outpatient procedure with a quick recovery and little risk of complications. On the other hand, subcutaneous mastectomy with primary reconstruction is a good option for selected women. This involves a combination of removal of the breast, and hence removal of the tumor, and in the same procedure the skin flaps are prepared for immediate breast reconstruction, either with a prosthesis or using autologous tissue [61]. The choice of technique requires a discussion with the patient prior to surgery. In this consultation, the patient needs to be well informed about the various procedures and what they may require from the patient. For example, reconstruction with prosthesis can be performed with a permanent prosthesis or with a tissue expander which then needs to be changed to a permanent prosthesis in a later procedure. However, the expander prosthesis can be gradually inflated to the final desired volume, while the permanent prosthesis has a specific size and shape which cannot be altered. The major advantage with a permanent prosthesis is the need for only one surgical procedure [62,63]. Both the surgeon and the patient should consider the pros and cons for both these options, and the surgeon needs to be prepared to make a final decision during the operation. There is another important aspect of implant-based reconstruction to consider, namely whether the implant is pre-pectoral or sub-pectoral (in front of or behind the pectoral muscle, respectively). Initially, all implants were sub-pectoral with a portion of the muscle covering and supporting the implant. There are few studies which directly compare pre- and sub-pectoral implant placement, since there are many components to the procedures, and they are therefore difficult to compare directly. A publication including 91 patients demonstrated a clear benefit in placing the implant behind the pectoral muscle [64]; however, this study dated from 1981 and both surgical techniques and surgical equipment have improved since then [65]. A more recent comparison (2018) reveals a superior outcome for pre-pectoral implant-based reconstructions [66]. The researchers reported less postoperative pain, faster recovery from postoperative upper extremity functional morbidity, and higher esthetic BREAST-Q scores as well as economic advantages in a series of 86 patients [66]. Proper selection of patients in the hands of an experienced surgeon is the main factor in achieving optimal outcomes and minimal complication rates. The final decision on pre- or sub-pectoral placement of the implant is taken by the surgeon during the surgical procedure [67].

Autologous reconstruction is a procedure which involves moving tissue flaps with an intact blood supply to the breast. This procedure takes significantly longer than the implant-based procedure, and often requires a longer hospital stay [63]. Many factors need to be considered when deciding on the optimal breast reconstruction technique, including patient age, body mass index, further oncological treatment required, patient's wishes, and obviously which methods are available [68,69]. A systematic review and a meta-analysis involving 219 studies comparing autologous versus implant-based reconstruction revealed significant differences in psychosocial and sexual well-being in favor of autologous reconstruction; however, there was no difference in physical well-being between the two groups [70].

Postmastectomy radiation therapy has a positive prognostic influence on LR and overall survival in breast cancer patients [71,72]. However, it is the biggest threat to implant-based reconstruction. Radiation therapy increases rates of infections, capsular contracture, implant loss, and overall reconstructive failure, which requires additional surgery [73,74]. In addition, reconstruction with a tissue expander prior to postmastectomy radiation therapy has a higher incidence of reconstructive failure compared to permanent silicone implants [75].

The oncological safety of performing a skin-sparing mastectomy (SSM) compared to a non-skin-sparing mastectomy has been shown in two large meta-analyses [76,77]. The first of these included nine studies with approximately 3700 patients [76]. The stage of disease was comparable in the mastectomy group and in the group of patients undergoing SSM. There was no significant difference in LR between the groups (6.2% vs. 4.0%, odds ratio (OR) = 1.25, 95% CI: 0.81–1.94). There were fewer cases of distant relapse in the mastectomy group (10.0% vs. 12.7%, OR = 0.67, 95% CI: 0.48–0.94) but this may be biased due to patient selection [76]. The second meta-analysis was published five years later and included 20 studies and approximately 5600 patients [77]. The risk difference in LR between the two groups was 0.4%, while the risk difference for all possible outcomes was not significant [77]. A more recently published review covers indications for SSM and complication rates between the surgical procedures [61]. As for the meta-analyses, although they emphasize the oncologic safety of the procedures, the results are dependent on the skills of the individual surgeons and close collaboration with plastic surgeons is recommended. The urgent need for prospective studies is stressed in all these publications.

There has been a major increase in the utilization of nipple-sparing mastectomy (NSM) [78]. Preservation of the nipple is considered safe and it has great impact on quality of life [61,[79], [80], [81]]. A recent review of 14 publications showed there was no statistically significant difference in five-year DFS and mortality between NSM and SSM with resection of the nipple. NSM had a partial or complete nipple necrosis rate of 15%, and a higher overall complication rate than SSM, but this was due to the rate of nipple necrosis in the first group [82]. However, it is emphasized that these procedures should be performed by experienced breast surgeons in collaboration with plastic surgeons [61]. An ongoing international NSM registry has been initiated by the European Society of Surgical Oncology (https://www.essoweb.org/eurecca-inspire/). The aim of this registry is to gain insight into treatment strategies for women undergoing NSM and immediate breast reconstruction. The results should provide solid evidence of the oncological safety of these procedures and will help to optimize patient satisfaction by using patient-reported outcome measures (PROMS). Neoadjuvant chemotherapy (NAC) is not a contraindication for NSM [83,84]. NSM after NAC is not associated with statistically significant differences in terms of post-operative complications, total nipple loss for necrosis, or involved margins, and results improve with the experience of the clinician. The locoregional relapse rate was higher after NAC, yet it was consistent with traditional mastectomy in this group of high-risk patients [83].

Malignant breast tumors in young patients are known to show aggressive behavior [85,86] with a significantly worse prognosis than for older patients [[87], [88], [89]]. It may be difficult to compare studies concerning young women, mainly because the definition of “young” differs between individual studies [90,91]. Due to aggressive tumor behavior, young women were previously recommended mastectomy rather than BCT, which was supported by the literature [92]. However, a reluctance to perform SSM in young women with aggressive tumor biology has been questioned. The oncologic safety in these patients was demonstrated in a retrospective study comparing SSM and immediate reconstruction with conventional mastectomy in women under 35 years [93]. The cohort consisted of 118 patients in the skin-sparing group and 141 in the group undergoing conventional mastectomy. After adjusting for tumor stage there was no statistically significant difference in disease-free survival and breast cancer-specific survival between the two groups [93].

There are numerous publications with robust data showing that BCT is non-inferior compared to mastectomy in terms of OS, contralateral breast cancer, distant metastases or second primary cancers [8,16,17]. Some of these even indicate that BCT is superior to mastectomy [8,94]. In most publications, locoregional recurrence is shown to be more frequent in patients treated with BCT [16,17]. However, this varies according to tumor biology [94]. Early stage triple negative breast cancer (TNBC) treated with modified radical mastectomy without adjuvant radiation had a significantly increased risk of locoregional recurrence compared to BCT followed by radiation [94].

Despite this knowledge there is an increasing trend towards mastectomy [95,96]. There is a similar trend towards contralateral prophylactic mastectomy (CPM) even though studies have confirmed that this is not associated with improved survival outcome [97]. This trend was mirrored in a decrease in unilateral mastectomy [97]. This increase in CPM, with a concomitant increase in breast reconstruction, is most evident in the United States, and the rise was noted across all ages, stage of cancer, racial groups, and geographic regions [97]. Factors influencing CPM were young age, white ethnicity, marital status, family history of breast cancer, use of hormonal replacement therapy, testing for BRCA1 and 2, higher tumor stage, lobular carcinoma, and the possibility of reconstruction [98].

### 2.3 Surgical treatment of the axilla in early stage breast cancer

The status of the axillary nodes is vital in predicting the outcome for patients with early stage breast cancer [99,100]. A study by the American College of Surgeons in 1978, which included 498 hospitals distributed over 47 states reported that the five-year survival rate was reduced from 60.5% in clinically localized disease (malignant disease in the breast where regional lymph nodes were not involved) to 49.1% in locoregional disease (malignant disease in the locoregional lymph nodes) [101]. This finding was confirmed in a later review involving 69 trials and more than 8000 patients [99]. The presence of axillary metastases decreases the patient's five-year survival by between 28% and 40% [101,102]. The role of sentinel lymph node dissection (SLND) and examination in breast cancer surgery is considered to be a safe procedure with few complications [103,104] and is reliable [100,104]. Axillary lymph node dissection (ALND) has previously been a standard procedure for staging of the axilla.

The National Surgical Adjuvant Breast and Bowel Project (NSABP) B-32 was a randomized controlled phase 3 trial performed in Canada and the USA between 1999 and 2005, which included 80 centers [105]. The trial confirmed that in patients with negative sentinel lymph nodes (SLN), the OS, DFS and regional disease control were equivalent in those who underwent SLN resection alone and those who underwent SLN resection plus ALND [105]. However, the latter procedure is associated with considerable arm morbidity, including lymphedema, sensory nerve damage, hemorrhage, and formation of seroma [106,107]. Long term consequences based on self-reported questionnaires confirm that this is a problem for a significant number of patients [108]. Chyle leakage as a complication of ALND has an incidence of less than 0.7% [109]; however, when it occurs it can be difficult to treat. SLND causes limited arm morbidity compared to ALND [103,106] and has, therefore, gradually replaced ALND as the standard procedure for staging of the axilla.

SLND allows extensive histopathological examination of the lymph nodes, with and without metastases [110,111]. Pathologically, there is a broad spectrum of clinical presentation for lymph node metastases. The mode of detection is either hematoxylin and eosin (H&E) and/or immunohistochemistry (IHC). The extent of the metastases is described using different parameters including size and potential growth beyond the lymph node capsule. The size of the metastases ranges from H&E-detectable macrometastases (defined as >2 mm) to H&E− and/or IHC-detectable micrometastases (≤2 mm), staged as N1(mi), to isolated tumor cells (≤0.2 mm) visualized via H&E and/or IHC staining and staged as N0(i+) [112].

The presence of macrometastases worsens the prognosis in breast cancer [99]. However, the presence and significance of micrometastases and/or isolated tumor cells (ITC) is questionable. In a study involving 109 patients with micrometastases in the sentinel nodes, the overall frequency of metastases in axillary non-sentinel nodes was 21,8% [113]. The frequency was significantly associated with the size of the micrometastatic lesion in the sentinel node. It varied from 44.7% in those cases approaching macrometastatic spread, to 15.6% in patients with micrometastases of <1 mm [113]. The conclusion from this study, and similar studies [114], was that patients with micrometastases in the SLN should continue to undergo ALND, while those with ITC should not [115]. However, this advice was later altered based on results from the International Breast Cancer Study Group (IBCSG) 23-01 multicenter, randomized, non-inferiority, phase 3 trial, with 5 and 10 years of follow-up [116,117]. In these studies patients with micrometastases to SLN were randomized to either undergo ALND or not to undergo ALND. 5-year disease-free survival was 87·8% (95% CI 84·4–91·2) in the group without axillary dissection and 84·4% (80·7–88·1) in the group with axillary dissection (log-rank p = 0·16; HR for no axillary dissection vs axillary dissection was 0·78, 95% CI 0·55–1·11, non-inferiority p = 0·0042). The findings of the IBCSG 23–01 trial after a median follow-up of 9·7 years corroborate those obtained at 5 years.

The possibility of minimizing morbidity following local therapy without negatively affecting outcome has been recognized and is supported by the results of recent trials. The American College of Surgeons Oncology Group (ACOSOG) Z0011 trial in 2011, with patients enrolled at 115 sites in the USA, demonstrated that with appropriate systemic therapy and radiation, clinically node-negative patients with positive sentinel nodes, who received breast-conserving surgery did not have an inferior outcome when complete ALND was omitted [118]. The AMAROS trial (published in 2014), which included almost 5000 patients enrolled at 34 centers from nine European countries, proved that complete ALND and axillary radiation after identification of a positive sentinel node were comparable in terms of local control for patients with tumors <5 cm without palpable axillary lymph nodes [119]. These studies have provided physicians with the confidence to spare patients the addition of complete axillary clearance while supporting the importance of other modalities, namely radiotherapy and systemic therapy, in optimizing breast cancer management.

However, the results of the ACOSOC-Z0011 study were questioned by specialists around the world. The three main concerns were: 1) follow-up was too short, especially considering the awareness of late recurrence in estrogen receptor (ER) positive patients; 2) specialists argued that the trial had involved a highly selected population and that the results were not applicable to all patients, and finally 3) the recommendations were not considered safe for high risk patients [120]. The first concern was addressed by a long-term follow-up study with significant results for non-inferiority of LR and OS in patients treated with sentinel node dissection alone versus complete axillary clearance [121,122]. The next two concerns, involving the selection of patients and the safety of high risk patients, were addressed by Mamtani et al. who looked at patients younger than 50 years old, and those with HER2 + and TN disease [123]. The group found that the need for complete axillary dissection in these patients was the same as for those with a more favorable tumor biology, and in the cases where a complete axillary dissection was performed, there was no greater burden of disease in the axilla (the number of positive nodes did not differ) [123]. This was later confirmed in a prospective study including almost 800 patients who all met the ACOSOC-Z0011 study's eligibility criteria [124]. The recommendations for surgery of the axilla in patients undergoing primary surgery are illustrated in Fig. 2.

The next step is to be able to select patients with clinically and radiologically lymph node-negative early stage breast cancer who can be spared any form of axillary surgery, including sentinel lymph node biopsy (SLNB), without impairing oncological safety. The issue was investigated in a retrospective study of 1360 patients with primary breast cancer, who underwent SLND, with or without ALND. The study evaluated tumor localization, multicentricity and multifocality, histological subtype, tumor size, histological grade, lympho-vascular invasion (LVI), HR status, and HER2 status. The presence of a large tumor or LVI were the only independent predictive factors of metastatic spread to the SLN [125]. The issue was further evaluated in a prospective study, the Intergroup-Sentinel-Mamma (INSEMA) Trial [126], but the results were not as convincing as in previous studies, which demonstrated that ALND could be safely omitted [118,119]. In the INSEMA Trial there was a significant degree of patient selection bias in terms of morphological differences in the control arm where SLND was omitted, but there was also a selection bias between the different centers as to which patients were assigned to NAC. The economic consequences of omitting SLND were not greater than performing the procedure [126]. A comparison of observation, axillary radiation, and complete axillary clearance for the management of the axilla in patients with a positive sentinel node was also addressed through a large systematic review of current trials [127]. This review identified almost 5000 publications, and after excluding various studies for different reasons, resulted in 10 trials being included in the narrative synthesis [[116], [117], [118], [119],^121^,^122^,[128], [129], [130], [131]]. Three of these studies compared observation with ALND [100,118,130], and two compared axillary radiation with ALND [119,128]. There was no significant difference in OS, DFS or axillary recurrence in these groups. Four trials registered morbidity outcome [100,117,119,131], and all concluded that ALND was associated with increased morbidity such as lymphedema, paresthesia, and shoulder dysfunction. Conclusively, the omission of complete axillary clearance in selected patients was considered safe and incurred significantly less morbidity [127]. These findings are in accordance with international guidelines used worldwide today [6,7,[36], [37], [38]].

### 2.4 Effect of neoadjuvant treatment on choice of surgical procedure

#### 2.4.1 Breast surgery after neoadjuvant treatment

NAC is the standard of care for patients with locally advanced breast cancer [6,7,36,37]. A pathologic complete response (pCR) is a positive prognostic factor with great impact on OS and recurrence-free survival [[132], [133], [134], [135]], especially in the most aggressive tumors [[134], [135], [136]]. Neoadjuvant treatment is routinely performed in all patients with a tumor size <5 cm. For patients with tumors between 2 and 5 cm the order of surgery and additional treatment is based upon the histopathological characteristics of the tumor, and treatment is often discussed by a multidisciplinary team. There is no difference in OS or DFS in patients receiving adjuvant treatment either pre- or postoperatively [137]. Until recently, these patients routinely had a mastectomy even if they had a pCR. Today, the aim is to perform BCT in patients with a radiologic complete response (rCR), but also in those cases with a partial response where it is still technically possible to perform BCT. There is no difference in local recurrence rate (LRR) in patients down staged to BCT and there is no difference in LRR after NAC with respect to the type of surgery [138,139]. A meta-analysis has shown that distant recurrence, DFS, and OS are better in patients who respond well to neoadjuvant treatment with breast conserving surgery as opposed to mastectomy [140]. There was no significant difference in RR or locoregional recurrence.

Assessment of the disease response to chemotherapeutic agents prior to any surgical intervention is also necessary as medical oncologists may tailor further treatment in ongoing regimens according to the response. Where there is no response, surgery may be performed earlier than initially planned. There is currently no standard imaging method for monitoring the response to therapy, but magnetic resonance imaging (MRI) seems to be the best option, with a reasonably high sensitivity (86%–92%, but a lower specificity (60%–86%) [141].

The next challenge is the extent of the resection. In some cases, the tumor may show clear concentric shrinking making it fairly easy for the surgeon to decide what to excise. Asymmetric shrinking, producing scattered residual enhancement on the MRI, makes it difficult to decide what is the actual tumor and what is necrotic disease from the preoperative medical treatment. MRI as a predictor of rCR [142] as well as pCR(143) varies between biological subgroups [[142], [143], [144]]. This awareness of the variation in tumor shrinkage and scattered residual disease led to an agreement between the American College of Radiology, the American College of Surgeons, the College of American Pathology, and the Society of Surgical Oncology concerning re-excision after neoadjuvant treatment that differs from the standard of care for BCT having primary surgery. If there is a viable tumor present throughout the specimen, even if it does not extend to the margin, a further re-excision should be considered [7].

Patients with calcification visible on the mammogram, multifocal multicentric lesions, invasive lobular cancer, or non-mass enhancement in pretreatment MRI, are significantly associated with false-negative results on MRI after NAC. The results for these patients should therefore be interpreted with caution [142]. In addition, luminal subtypes are associated with a high false negative rate (FNR) when evaluating rCR after NAC.

Post-neoadjuvant systemic treatment (NST), residual mammographic microcalcifications have a lower correlation with residual tumor size than enhancing lesions on MRI. Other than in patients with an HR+/HER2-subtype, the extent of calcifications during preoperative evaluation is not considered to be accurate in predicting the extent of the residual tumor after NST [144].

It is mandatory to mark the tumor before starting chemotherapy in order to be able to perform BCT [145]. There are strict criteria for BCT after neoadjuvant treatment. The primary tumor bed must be localized either by residual mass, calcification, or previously inserted radiopaque clips. In addition, there must be an acceptable tumor-volume to breast-volume ratio and an absence of diffuse suspicious microcalcification. Multicentricity, either at presentation or after NAC, is a matter of concern, but is not an absolute contraindication [146]. It is also important to know that there is an increased risk of lumpectomy failure in cases of invasive lobular carcinoma [145,147] as opposed to other histological tumor types. Furthermore, it is important to keep in mind that the pCR rate varies depending on the biological subtype and therapy used [[148], [149], [150], [151], [152], [153]]. Post-treatment change in the proliferation marker Ki67 after NAC is used as a marker of treatment response and is therefore associated with improved survival [153]. Patients with triple negative disease have significantly higher pCR rates than those with HR-positive disease. With adequate targeted treatment, HER2 enriched cancers have the greatest pCR rates, especially when a dual anti-HER2 blockade is applied [148,149].

To conclude, patients undergoing BCT after NAC have an excellent five-year locoregional recurrence-free survival with variable responses according to molecular subtype and response to NAC [154]. The histological subtype is relevant when choosing patients who are eligible for BCT after neoadjuvant treatment. In a large meta-analysis including 17 studies, there was a significant difference in the pCR rate between ductal carcinoma and lobular carcinoma (5.9%–16.7%; OR = 3.1, 95% CI: 2.48–3.87, P < 0.00001), while the OR for having a breast conserving surgery was significantly higher in ductal carcinomas (35.4%–4.8%; OR = 2.1, 95% CI: 1.8–2.45, P < 0.00001) [147].

As mentioned previously, multicentricity is not an absolute contraindication for BCT after neoadjuvant treatment [146]; however, it is a matter of concern. In a study of more than 6000 patients, the tumors were divided into unifocal, multifocal or multicentric. Those patients with multicentric tumors had worse DFS (P < 0.001) and OS (P = 0.009) than patients with unifocal tumors. However, local recurrence-free survival (LRFS), DFS, and OS were not inferior for patients with multicentric or multifocal tumors if pCR was achieved. Tumor-free margins are naturally required [146]. This means that in selected patients with multifocal or multicentric breast cancer, BCT with a wide resection is not associated with inferior local disease control and can be considered when acceptable cosmetic results can be achieved [146,155].

The omission of breast cancer surgery entirely in complete responders to neoadjuvant therapy has also been questioned. This was addressed in a single-center prospective study including 40 patients, all with triple-negative or HER2+cancers, and a TNM (Tumor Node Metastasis) status of T1-3N0-3 [156]. Approximately half of these patients (47.5%) had a breast pCR, indicating no residual invasive or *in situ* changes in the breast. The radiological response in these 19 patients with pCR was surprisingly not complete; 12 of the 19 (63.2%) had both pCR and rCR. Image-guided biopsies (both fine needle aspiration and vacuum-assisted core biopsy) correctly identified the patients with pCR in 39 out of the 40 patients (97.5%). Importantly, these 39 patients had a concordant breast pathologic response and pathologic nodal status. The remaining patient had only micrometastases in the sentinel node. This correlation between breast pCR and nodal pCR agrees with results from a retrospective study of 237 patients with biopsy-proven positive lymph node disease [157]. In the same study, all 116 patients with breast pCR also had axillary pCR. This opens the way for a prospective clinical trial where breast surgery can be omitted, and this has already been initiated at the University of Texas M.D. Anderson Cancer Center, starting in January 2017 and with estimated completion in January 2022 [158].

#### 2.4.2 Surgery of the axilla after neoadjuvant treatment

Sentinel node biopsy can be performed safely on patients receiving neoadjuvant therapy and this is the standard of care for clinically node-negative patients prior to chemotherapy [159,160]. Neoadjuvant treatment not only downstages the breast tumor but also downstages disease in the axilla [156,157,161], and as in the breast, pCR of the axilla is associated with a significant prognostic benefit [153]. Patients with a biopsy-proven positive lymph node have, up until recently, routinely been treated with complete axillary dissection. It has been reported that neoadjuvant treatment changes patient status from clinical node-positive to clinical node-negative in 35%–49% of cases [123,[161], [162], [163]]. There are five possible histological outcomes after NAC: 1) no change (clinical stage is the same as the pathological stage post-NAC); 2) breast-only pCR; 3) node-only pCR; 4) overall pCR (breast + axilla) or 5) upstage of disease (pathological state post-NAC worse than clinical stage) [164]. However, a sixth possibility involves a partial response in the node, breast, or both.

A review of breast cancer patients from the National Cancer Data Base (NCDB) included women with cT1–3/cN0–1 breast cancer diagnosed between 2010 and 2014 who underwent surgery following NAC. Approximately 33 000 patients were identified, and after exclusion of patients with discordant or partial post-NAC response, around 20 000 were evaluated further [164]. The patients were divided into four groups based on their HR and HER2 status: HR+/HER2-, HR+/HER2+, HR-/HER2+, and triple negative (TN). Based on the different histological outcomes, 19.2% experienced overall pCR, 1.5% breast-only pCR, 3.4% node-only pCR, and 29.1% no change, with 7.9% experiencing tumor upstaging. The different outcomes and subtypes were evaluated with respect to OS and it was found that in node-positive patients, pCR when limited to either the breast or axilla predicted survival for selected receptor subtypes. In patients achieving pCR in both the breast and axilla, survival is driven by response to NAC rather than the clinical status of the lymph node [164]. With the implementation of the results from the ACOSOC- Z0011 study, of de-escalating surgery of the axilla in early breast cancer, it has been tempting to de-escalate surgery in the axilla of those patients who have become clinically node-negative after NAC. Both the safety and reliability of the SLN procedure in these patients are of importance.

Three prospective studies have addressed this matter and have established the FNR for SLN in this setting: the ACOSOG Z1071 (Alliance) Prospective Multicenter Clinical Trial [162], the SN FNAC study [163], and the SENTINA study [165]. Two of these studies had an FNR below 10%, which is acceptable for primary surgery [162,165]. However, the FNR is closely related to the number of SLNs removed. If more than 3 nodes are removed the FNR is below 10%. When only one or two nodes are removed, the FNR is above 10%, which is not acceptable. When IHC is included in the evaluation of the SLN procedure, a further decrease in the FNR is obtained, and therefore IHC is required in these cases [162,163].

The next concern was if the SLN was the same lymph node as the biopsy-proven metastatic lymph node detected prior to neoadjuvant treatment. It was reported that ultrasound and palpation of the axilla following neoadjuvant therapy were not accurate enough, and that additional tools and/or imaging were needed [166]. The ACOSOG Z1071 study addressed this question by placing a clip in the biopsy-proven metastatic node at the time of diagnosis [167]. In 75.9% of cases, the clipped node was within the SLN. The application of targeted axillary dissection (TAD) of the clipped node in addition to the combination of dual tracer, removal of at least two SLNs, and clinical selection of patients through axillary ultrasound, led to a reduction in the FNR to 6.8% [167]. There are various alternatives for marking an affected lymph node, identifying and surgically removing it. The initial study used a metallic clip marked with a guide wire prior to surgery. Confirmation of clip removal through radiological examination of the surgical specimen was necessary [167]. The affected lymph node can also be marked with a radioactive seed, which is identified by the surgeon per-operatively using a gamma probe [168]. The latter procedure is preferred by most surgeons because it involves the same technique used in SLN detection and only requires changing the probe setting.

There are alternative options for preoperative marking of the SLN, a biopsy-proven affected lymph node, as well as a tumor in the breast itself. The SentiMag® magnetic localization system is a procedure based on the detection of a magnetic particle, which is placed in the lymph node or breast lesion. This is identified with a handheld magnetometer (SentiMag®). A meta-analysis including five clinical trials comparing this method to standard methods of detecting SLNs confirmed its non-inferiority [169]. SAVI SCOUT® is another potential method based on a non-radioactive infrared-activated electromagnetic wave reflector. The reflector remains passive until activated using the manufacturer's console and handpiece system. A pilot study using this method performed on 50 patients confirmed that it was safe and effective for guiding the excision of non-palpable breast lesions [170]. The evaluation of longer duration use of the SAVI SCOUT® system has been tested in a pilot study with neoadjuvant treated patients (NCT03015649, CMI- SCOUT-001). The trial was initiated in 2017 and has been completed but the results have not yet been published. The SAVI SCOUT® technique was FDA-approved in 2014, and SentiMag® was approved in 2016. A brief summary on the alternatives to standard pre-operative localization of non-palpable breast lesions was published by Jeffries et al., in 2017 [171].

Through prospective trials, the safety and utility of surgery of the axilla in neoadjuvant-treated patients has been confirmed both in 1) patients with clinically node-negative disease prior to neoadjuvant treatment and 2) patients with clinically node-positive disease prior to neoadjuvant treatment. The procedure itself requires removal of at least two lymph nodes, application of IHC in addition to H&E, and confirmation that the removed lymph node is the biopsy proven metastatic lymph node. When these requirements are followed, the procedure is considered be reliable. Current recommendations suggest that if the SLN is negative at surgery, no further dissection of the axilla is needed. If the SLN is positive, the surgeon then proceeds with complete axillary dissection. If the SLN is not localized a complete axillary dissection is advised [6,7,37]. The concept of clipped/marked nodes (TAD) has not yet been introduced in all surgical units around the world [6]. The development of surgery in the axilla, and the confirmation of the safety and utility of the SLN procedure in neoadjuvant-treated patients has been well reviewed by Fisher et al. [172]. The recommendations for surgery in the axilla in neoadjuvant-treated patients are illustrated in Fig. 3.

The next important question is whether or not regional nodal irradiation (RNI) improves the recurrence-free interval in patients that are biopsy-proven lymph node-positive prior to NAC and who, after treatment, become pathologically node-negative. A further question is whether patients who remain node-positive after NAC can be spared ALND if they receive RNI in addition to axillary radiotherapy. These two issues have been addressed in two randomized trials, the NSABP B-51/RTOG 1304 trial [173], and the Alliance A11202 trial [174]. The estimated completion dates are 2028 and 2024, respectively.

During this era of de-escalating surgery, it is important to stress that breast cancer is a heterogenous disease [175]. A one-size-fits-all solution is clearly not a viable approach. Considering tumor biology is crucial in all steps, and especially in the response to neoadjuvant therapy [[176], [177], [178], [179]]. HER2-overexpressing tumors and TN tumors respond well to neoadjuvant treatment, and in stage 2 or 3 of HER2+ or TN disease, NST is the preferred initial approach [38,156] enabling these patients to avoid axillary dissection and making them possible candidates for BCT [38].

### 2.5 Quality of life in breast cancer survivors

It has been established that oncoplastic breast surgery is safe [15], but is there a difference in esthetic outcome? Patient-reported esthetic and functional outcomes after conventional and oncoplastic resection have been evaluated [180] and it seems that esthetic outcome after conventional resection is as good as with oncoplastic surgery with the right selection of patients. Oncoplastic resection enables BCT in patients with larger tumors and those with multifocal tumors with a favorable esthetic outcome [180]. These are women where BCT is not an option without tumor reduction through neoadjuvant chemo- or endocrine therapy. A recent review by Cardoso et al. [181] addressed the question of how the esthetic outcome has changed over the years and, more importantly, investigated methods of training and recommendations for future efforts in achieving the best possible esthetic outcome [181]. With an increasing number of breast cancer survivors, there is an increase in the number of women living with an unsatisfactory esthetic outcome and quality of life studies clearly shows that this adversely affects the patients. There are not only esthetic concerns, but also issues with chronic pain, and cognitive and sexual changes that lead to a decreased quality of life [182]. How to evaluate the esthetic outcome is a challenge, but there are methods available, both subjective [183,184], and objective, as evaluated by a surgical expert [185], and finally objective protocol-based [186] methods. None of these are perfect, and for an optimal surgical outcome, the centralization of breast cancer surgery may be a solution [57].

Even though these factors were not considered in the early days of breast cancer treatment, it has become clear that quality of life, body image, and psychosocial well-being are critically important to women after mastectomy [187]. Breast reconstruction offers significant benefits when considering quality of life in these women [[188], [189], [190], [191], [192], [193]]. The techniques for reconstruction after a mastectomy, whether primary or secondary, may naturally vary. The major differences are implant-based breast reconstruction as opposed to autologous breast reconstructions. A large multicenter study from Michigan, USA compared two-year complication rates associated with common techniques for postmastectomy breast reconstruction among 2343 women registered at 11 sites participating in the Mastectomy Reconstruction Outcomes Consortium study [194]. The results revealed high rates of overall complications and re-operative complications, with significantly higher odds of complications associated with autologous reconstruction compared with implant-based techniques. However, although failure rates were low across procedure types they were higher in the implant-based reconstructions. Delayed reconstructions were significantly less likely to develop any complications compared with women receiving immediate reconstructions.

Using data from the same multicenter study (which involved 57 plastic surgeons), colleagues from Michigan used the BREAST-Q survey to examine patient satisfaction and breast-related quality of life two years after breast reconstruction using implant or autologous techniques [195]. After stratification for baseline patient characteristics, it was found that patients who underwent autologous reconstruction had greater satisfaction with their breasts, and improved psychosocial and sexual well-being at two years compared with patients who underwent implant-based reconstruction [195]. To our knowledge, both these studies [194,195] represent the largest prospective multicenter, patient-focused outcome series on breast reconstruction. However, before firm conclusions can be drawn, further long-term (>10 years) analyses of longitudinal and cost-effective outcomes in a similar cohort must be performed. We know that there are more complications with reconstructive surgery compared to conventional mastectomy and it is important to inform the patients about these possible complications so that they can make an informed decision on which procedure is best for them [62,196,197].

The choice of mastectomy with implant-based reconstruction may seem like a good solution, but it is not the best choice for all. It is important to bear in mind how the patients themselves experience the different surgical procedures. By using the BREAST-Q patient-reported outcome measure, BCT was compared to mastectomy with implant-based reconstruction in a study involving approximately 3200 patients. Of these women, 63% had BCT, 4% had nipple-sparing mastectomy and 34% had skin-sparing or conventional mastectomy [198]. Baseline characteristics like age, marital status, race, body mass index and clinicopathologic characteristics of the tumor were included in the evaluation, and overall patients with BCT were most satisfied. This knowledge is important and may be of help in counseling patients. The International Consortium for Health Outcomes Measurement (ICHOM) organized a multidisciplinary working group for breast cancer with the intention of providing a minimal standard set of outcomes for patients with breast cancer [199]. The aims of the group were to: 1) enhance clinician-patient shared decision-making, 2) provide quality outcome information to providers and institutions to drive transparency and improvement, and 3) increase the opportunity for comparative effectiveness research [199].

The economic consequences of different levels of oncoplastic surgery is another aspect of breast cancer treatment. Cost-utility analyses from the USA, where oncoplastic surgery is most widespread, have been performed. They compared large volume displacement oncoplastic surgery to mastectomy with implant-based reconstruction [200] and free flap reconstruction [201] in the treatment of breast cancer. In both cases, oncoplastic BCT was found to be more cost effective [200,201].

### 2.6 Quality control of surgical procedures

Quality control of surgical procedures is important both for the patients and for the different clinics. The National Surgical Quality Improvement Program (USA) conducted a study looking at reoperation for complications after breast conserving surgery and mastectomy [202]. It included 18 500 patients. Only 4% required an unplanned reoperation within 30 days, and the most frequent operation was mastectomy with immediate breast reconstruction. Bleeding is the most common complication requiring reoperation [202].

To maintain the excellent results for breast cancer treatment that we have today, there must be general rules and requirements for treatment both internationally [35,54,55,59] and nationally [203]. Included in this concept are requirements that must be met by the breast cancer centers treating patients as well as strict requirements on quality control [35,54,55,59,203]. This includes the clinicians involved, both oncological surgeons and medical oncologists, surgical skills, medical equipment (pharmaceutical and technical), the number of patients treated in the unit, research activity, and the proper use of multidisciplinary teams [35,56,57].

## 3 Conclusions

Breast cancer is a heterogenous disease which unfortunately affects a significant number of patients, mostly women but also some men. Because of its heterogeneity a “one-size-fits all” treatment is not the correct approach. Information about breast cancer is available on-line and breast cancer patients are often well informed about their disease; however, even though the information is available to the public, it does not mean that it can be readily understood by those without a medical education and it is important therefore, for the physicians to be well prepared for the consultation. In addition, the different surgical procedures require efforts from the patient, both in terms of experienced pain and restrictions in daily routines. It is important for the clinicians to inform the patients in an understandable way that the chosen treatment is safe, and the patient is confident with the solution selected. Achieving the best possible treatment for breast cancer patients is considered the major goal for the health care system. This implies optimizing health outcomes per dollar spent and needs to encompass overall disease control, possible complications, and quality of life. There are many treatment options which can lead to the same surgical and oncological results, and many of these are decisions that need to be taken by the patient through informed consent.

To conclude, it is clear that the treatment of breast cancer is a field that is undergoing continuous change and improvements are occurring constantly. It is mandatory for the clinicians to be cognizant of, and up to date with, all these changes in order to be able to offer the best possible treatment. Fortunately, many patients diagnosed with breast cancer will outlive their cancer, which means the choice of optimal treatment will be crucial in terms of prognosis and quality of life.

## Ethical approval

This is a review where individual patients are not involved. Ethical approval is not relevant.

## Sources of funding

This manuscript is written without external funding. Publishing charges will be done through the medical library at Oslo University Hospital.

## Author contribution

MR has initiated the idea of the review and written the manuscript. ES and TS has critically read through the manuscript and added useful information which is added to the initial draft. Special thanks to Sarah Lang for help with language editing.

## Trial registry number

Name of the registry:

Unique Identifying number or registration ID:

perlink to your specific registration (must be publicly accessible and will be checked):

## Guarantor

Dr Margit Riis take the full responsibility for the work and the content of the review.

## Provenance and peer review

Not commissioned, externally peer-reviewed.

## Declaration of competing interest

There is no conflict of interest.
